# Identification of an Immune-Related Signature Predicting Survival Risk and Immune Microenvironment in Gastric Cancer

**DOI:** 10.3389/fcell.2021.687473

**Published:** 2021-11-02

**Authors:** Shuang Dai, Tao Liu, Xiao-Qin Liu, Xiao-Ying Li, Ke Xu, Tao Ren, Feng Luo

**Affiliations:** ^1^Department of Medical Oncology, Lung Cancer Center, West China Hospital, Sichuan University, Chengdu, China; ^2^Department of Oncology, The First Affiliated Hospital of Chengdu Medical College, Chengdu Medical College, Chengdu, China; ^3^Department of Oncology, Jintang First People’s Hospital, Chengdu, China; ^4^Department of Abdominal Cancer, Cancer Center, West China Hospital, Sichuan University, Chengdu, China

**Keywords:** gastric cancer, immune signature, tumor mutation burden, immune infiltration, survival

## Abstract

**Background:** Tumor immune microenvironment plays a vital role in tumorigenesis and progression of gastric cancer (GC), but potent immune biomarkers for predicting the prognosis have not been identified yet.

**Methods:** At first, RNA-sequencing and clinical data from The Cancer Genome Atlas (TCGA) were mined to identify an immune-risk signature using least absolute shrinkage and selection operator (LASSO) regression and multivariate stepwise Cox regression analyses. Furthermore, the risk score of each sample was calculated, and GC patients were divided into high-risk group and low-risk group based on their risk scores. Subsequently, the performance of this signature, including the correlation with overall survival (OS), clinical features, immune cell infiltration, and immune response, has been tested in GC data from TCGA database and Gene Expression Omnibus (GSE84437), respectively.

**Results:** An immune signature composed of four genes (MAGED1, ACKR3, FZD2, and CTLA4) was constructed. The single sample gene set enrichment analysis (ssGSEA) indicated that activated CD4^+^/CD8^+^ T cell, activated dendritic cell, and effector memory CD8^+^ T cell prominently increased in the low-risk group, showing relatively high immune scores and low stromal scores. Further GSEA analysis indicated that TGF-β, Ras, and Rap1 pathways were activated in the high-risk group, while Th17/Th1/Th2 differentiation, T cell receptor and PD-1/PD-L1 checkpoint pathways were activated in the low-risk group. Low-risk patients presented higher tumor mutation burden (TMB) and expression of HLA-related genes. The immune-associated signature showed an excellent predictive ability for 2-, 3-, and 5-year OS in GC.

**Conclusion:** The immune-related prognosis model contributes to predicting the prognosis of GC patients and providing valuable information about their response to immunotherapy using integrated bioinformatics methods.

## Introduction

Gastric cancer (GC) is a common digestive tract tumor and the third leading cause of cancer-related deaths (Bray et al., 2018). Although there have been advances in early screening and current therapies, the treatment and survival for GC remain unsatisfactory. The 5-year survival rate for GC is still lower than 30%, and survival for metastatic GC remains below 2 years (Ferlay et al., 2015). Further biomedical research is urgently recommended for screening novel diagnostic biomarkers and therapeutic targets.

Tumor immune microenvironment (TIM) is closely related to the tumorigenesis and tumor progression, as well as resistance to immunotherapy (Bindea et al., 2013; Quail and Joyce, 2013; Fridman et al., 2017). Accumulating studies have highlighted that heterogeneity exists in the proportion of intratumoral immune cell populations, and researchers try to identify those patients with specific immune response (Zeng et al., 2019; Zhang et al., 2019). The oncologists focus on activating immune responses, screening highly specific immune biomarkers, and exploring immune regulatory mechanisms to restore dysregulated immune microenvironment and improve survival outcomes (Eso and Seno, 2020). For example, the development of immune checkpoint inhibitors (ICIs) (Tang et al., 2018) has proposed the predictive biomarkers including microsatellite-instability (MSI) status, PD-1/PD-L1 expression, and tumor mutation burden (TMB) (Lee et al., 2016; Samstein et al., 2019). However, there is still no effective biomarker to predict sensitivity or resistance to ICIs. It is far from enough to fully understand the roles of tumor immune landscape in GC.

Bioinformatics analysis has been widely employed to screen prognostic signatures (Rooney et al., 2015; Jiang et al., 2018). With the help of ssGESA and CIBERSORT algorithms, some studies estimated the immune cell subsets and immune scores in order to gain a better understanding of the immune infiltration status and treatment response of immunotherapy in GC (Bindea et al., 2013; Newman et al., 2015). However, there has been no appropriate study that constructed a robust signature to predict immune status and the survival of GC based on immune-related genes, or could systematically evaluate the relationship between immune genomic characteristics and TIM (Shi et al., 2018; Zeng et al., 2019). Therefore, our study took advantage of TCGA and GEO database to determine key immune-associated genes and build a reliable signature using Lasso and multivariate Cox regression analyses. In addition, this signature further examined the predictive effect on the response of ICIs.

## Materials and Methods

### Data Download and Preprocessing

**FIGURE 1:**
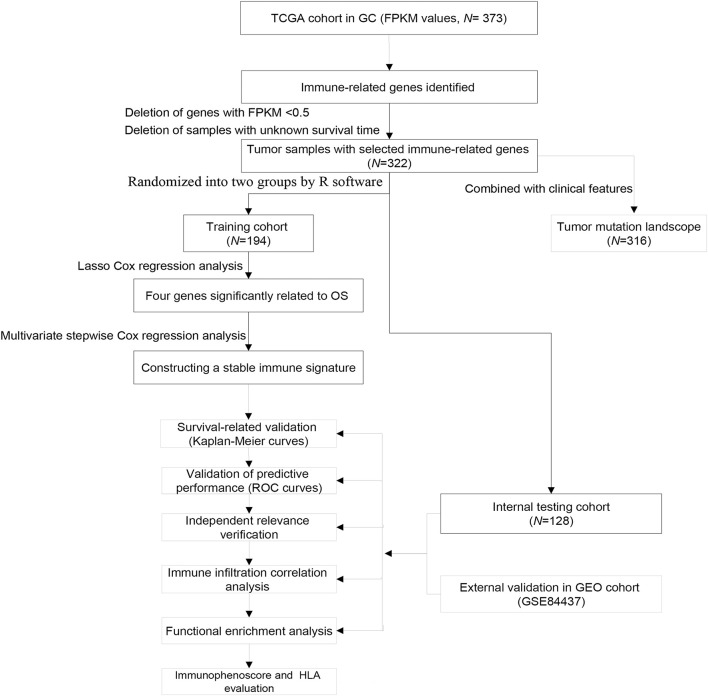
Flowchart of the study.

The FPKM values of gene expression profiles (*n* = 373) and corresponding clinical information were derived from the TCGA database (December, 2020), including 343 GC samples and 30 adjacent non-tumor samples. Tumor samples with the corresponding clinical data were randomized into training group and internal testing group, and then the data with unknown survival time and 0-month survival time were deleted. The immune-related gene profile is derived from the ImmPort database (Bhattacharya et al., 2014) and previous publications. GSE84437 cohort including 433 samples was also downloaded from the GEO database as an independent external validation group. The study did not need the approval from the ethics committees because all data were open-access in the TCGA or GEO database. The detailed flowchart of analysis steps is shown in Figure 1.

### Identification of Immune-Related Genes and Development of Immune-Related Risk Signature

Based on the ImmPort Database^1^ and the previous studies (Li et al., 2017; Gao et al., 2020; Sun et al., 2020), a total of 3,265 immune-related genes (IRGs) were collected. Then, after the deletion of low-expression genes (FPKM < 0.5), these IRGs were included into the Lasso analysis to integrate multiple variables. Lasso Cox regression analysis, a penalty regression tool, can estimate the regression coefficients by maximizing the log-likelihood function (or sum of squared residuals). Through Lasso regression analysis, the high-dimensional data were resampled 1,000 times, which can automatically delete unnecessary covariates and screen out the most stable prognostic factors. We used multivariate stepwise Cox regression analysis (forward and backward) to evaluate the capabilities of immune-related signatures in predicting OS of GC patients. Eventually, we calculated the total risk score of each patient (β genes × expression level of genes) in the training and validation cohorts by integrating the regression coefficients and gene expression levels, respectively. Based on the risk score, patients’ survival status, risk score levels, and heat maps of the immune signature were also presented. Subsequently, the samples were separated into high- and low-risk groups based on the median risk score of the training set. The predictive capacity of immune-risk signature was tested by Kaplan–Meier survival analysis using the *R* package “Survminer.” Time-dependent receiver operating characteristic (ROC) curves with AUC values were used to evaluate the ability of prognosis classification. Moreover, the independent predictive ability of the immune-risk signature also needed to be further assessed by multivariate analyses compared with other clinical factors including age, grade, and stage treated as continuous variables.

### ssGSEA Validation of Risk Signature Related to Immune Cell Infiltration

To further elucidate the biological understanding of immune-related riskScore, ssGSEA was applied to calculate the proportion of immune cells in the TIM of GC based on the comparison of GC expression profiles with the gene sets (Subramanian et al., 2005; Bindea et al., 2013). The gene sets comprising 782 genes were utilized to identify 28 types of immune cells and predict the abundance of corresponding immune cells. These types of immune cells were defined as three major immunocyte types including anti-tumor immunocytes (activated CD4^+^ T cells, activated CD8^+^ T cells, activated dendritic cells, CD56 bright natural killer cells, central memory CD4^+^ T cells, central memory CD8^+^ T cells, effector memory CD4^+^ T cells, effector memory CD8^+^ T cells, natural killer cells, natural killer T cells, type-1 T helper cells, and type-17 T helper cells), pro-tumor immunocytes (CD56 dim natural killer cells, myeloid-derived suppressor cells [MDSC], immature dendritic cells, neutrophils, plasmacytoid dendritic cells, macrophages, regulatory T cells, and type-2 T helper cells), and intermediate immunocytes (activated B cells, eosinophils, gamma delta T cells, immature B cells, mast cells, memory B cells, monocytes, and follicular helper T cells). We also compared the proportion differences of tumor immune cell infiltration between low- and high-risk immune groups by using Mann–Whitney–Wilcoxon tests.

### Correlation Analyses of Immune Response

Human leukocyte antigen (HLA) genes have a critical function in immune surveillance and response. The human MHC encodes a glycoprotein known as HLA, which plays an important role in T-cell antigen presentation (Mosaad, 2015; Dendrou et al., 2018). The MHC is divided into three subclasses: the class I region includes the classical gene types (HLA-A, HLA-B, and HLA-C genes), as well as the non-classical types known as MHC Ib (HLA-E, HLA-F, and HLA-G genes); the class II region includes the HLA-DPA1, HLA-DPB1, HLA-DQA1, HLA-DQA2, HLA-DQB1, HLA-DQB2, HLA-DRA, HLA-DRB1, HLA-DRB2, HLA-DRB3, HLA-DRB4, and HLA-DRB5 genes as well as less variable genes involved in antigen processing and presentation; and the class III region does not encode any molecules for antigen delivery and peptide binding, but contains genes only involving in inflammatory responses, leukocyte maturation and the complement cascade (Mosaad, 2015). We compared the expression of HLA-related genes among patients in different immune risk groups. In addition, Immunophenoscore (IPS), as a scoring tool for evaluating the tumor immunogenicity, was applied to analyze the correlation between the new immune signature and intratumor immune response. ESTIMATE algorithm was also utilized to calculate the Tumor Purity rate, ESTIMATE Score, Immune Score, and Stromal Score within the tumor microenvironment of GC in the TCGA and GEO groups, respectively (Yoshihara et al., 2013). The difference of immune scores and stromal scores among patients in different immune risk groups was expressed by “ggplot2” and “ggsignif” (*R* package).

### Functional Enrichment Analysis

The *R* package “limma” was used to analyze differential expression between high-risk and low-risk groups, and all genes were ranked by foldchange values. Gene Set Enrichment Analysis of KEGG was performed to clarify the significant annotated pathways (*P* < 0.05) through *R* package “gseKEGG of clusterProfiler” (Subramanian et al., 2005).

### Tumor Mutation Burden Analysis

The somatic mutational annotation file of GC in the TCGA was downloaded from Genome Data Commons. TMB, defined as non-synonymous somatic mutations per Mb, was counted by the total number of mutations in six mutation categories, including all GC splice site mutations, missense and non-sense mutations, in-frame insertions, frameshift mutations, and deletions. The *R* package “GenVisR” was conducted to investigate the relationship between the immune signature and TMB. Meanwhile, we performed multivariate analyses again to assess the predictive performance of the immune-risk signature by comparing with other factors including age, grade, stage, TMB, and IPS.

### qRT-PCR Analysis

A tissue microarray containing 13 independent STAD tissue samples and paired adjacent non-cancerous tissue samples was purchased from Shanghai Outdo Biotech Co., Ltd. (cat. no. cDNA-HStmA030CS01, Shanghai, China), and used to investigate the expression of four genes (MAGED1, ACKR3, FZD2, and CTLA4). The forward primer sequences were as follows: ACKR3-F: 5′- GACGCTTTTGTTGGGCATGT-3′ and the reverse primer sequence was ACKR3-R: 5′- ATT TGATTGCCCGCCTCAGA-3′ and the product length was 149 bp; FZD2-F: 5′- CTCCGTCCTCGGAGTGGTTC-3′ and the reverse primer sequence was FZD2-R: 5′- GCGAAGC CCTCATGAACAAG-3′ and the product length was 117 bp; MAGED1-F: 5′- AGGTCTGCATAAGCAAGGCG-3′ and the reverse primer sequence was MAGED1-R: 5′- TGCTGC CTTCTTCGTCAAGC-3′ and the product length was 143 bp; CTLA4-F: 5′- GCAGCAGTTAGTTCGGGGTT-3′ and the reverse primer sequence was CTLA4-R: 5′- CTCTGTTGGGGG CATTTTCAC-3′ and the product length was 123 bp. The primers of β-actin (reference gene): forward: 5′-GAAGAGCTACG AGCTGCCTGA-3′ and reverse: 5′- CAGACAGCACTGTGTTG GCG-3′. All RT-PCR operations were performed according to the manufacturer’s instructions. In short, a 20-μl mixture containing 10 μl of TB Green Premix Ex Taq (Tli RNaseH Plus) (TaKaRa Bio Inc., Japan), 1 μl of 100 μM primer mix (Tsingke Biotechnology Co., Ltd., Beijing, China) and 9 μl of RNase/DNase-free sterile water were added to each cDNA sample, and then the cDNA arrays were sealed with a transparent sealing film, and placed on ice for 15 min. The qRT-PCR reaction was carried out in the LightCycler 96 Real-Time PCR System (Roche Diagnostics, Indianapolis, Ind.). The reaction mixture was activated at 50°C for 2 min, pre-denatured at 95°C for 10 min, followed by 40 cycles of amplification reaction at 95°C for 15 s and 60°C for 1 min. Finally, LightCycler 96 software (Version 1.1.0.1320, Roche) was used for the collection and analysis of qRT-PCR data. The relative mRNA expression level was calculated by the 2^–ΔΔCt^ method taking β-actin as the reference gene.

### Statistical Analysis

*R* statistics software (version 3.6.1) was used for all statistical analyses. The chi-square test for qualitative variables was used to estimate the differences between groups. Differences between two or more than two cohorts were examined by non-parametric tests including Mann–Whitney–Wilcoxon test and Kruskal–Wallis *H* test in quantitative data. Chi-square test, Mann–Whitney–Wilcoxon test, and Kruskal–Wallis *H* test were performed with the *R* functions “chisq.test,” “wilcox.test,” and “kruskal.test,” respectively. *P* values less than 0.05 were considered to be statistically significant.

## Results

### Construction of Immune Risk Signature

**FIGURE 2:**
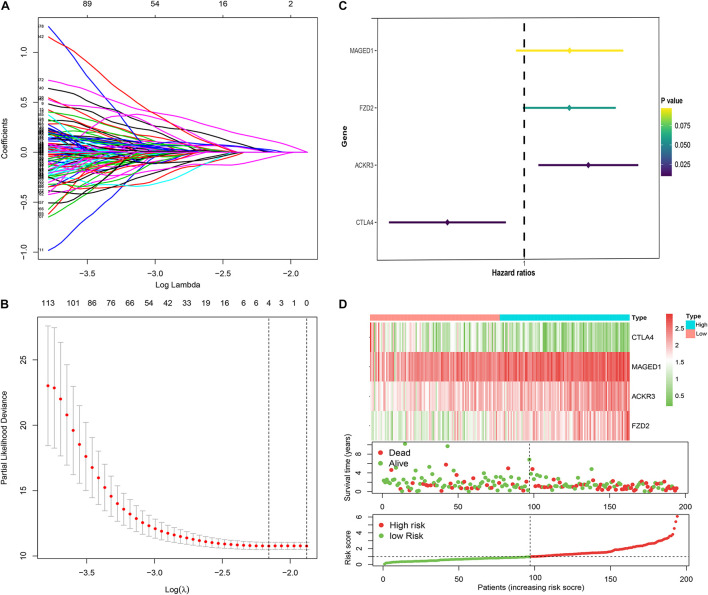
Construction of the immune risk signature model. Lasso regression analysis **(A,B)** and multivariate stepwise Cox regression analysis **(C)** for identification of the immune risk signature; expression heat map (upper), survival status (middle), and risk score (bottom) of the signature consisting of four immune-related genes were depicted **(D)**.

A total of four immune genes (MAGED1, FZD2, ACKR3, and CTLA4) were screened out through Lasso regression analysis (Figures 2A,B). In order to strictly examine their prognostic significance and establish an immune-risk signature, these four genes were further included into multivariate stepwise Cox regression analysis, and a prognostic Cox regression model was established (Figure 2C). Based on regression coefficients and expression levels, the formula of risk score was as follows: (0.2615 × expression level of MAGED1) + (−0.3691 × expression level of FZD2) + (0.2597 × expression level of ACKR3) + (−0.4425 × expression level of CTLA4). The patients were divided into high- and low-risk cohorts according to the median value of the training group. Based on the risk score, patients’ survival state, risk score levels, and heatmaps of this signature in the training group are shown in Figure 2D in detail. Validations in the TCGA and GEO sets are shown in Supplementary Figure 1. Taken together, these genes are most likely to participate in GC.

### Validation of Prognostic Value of Risk Signature

**FIGURE 3:**
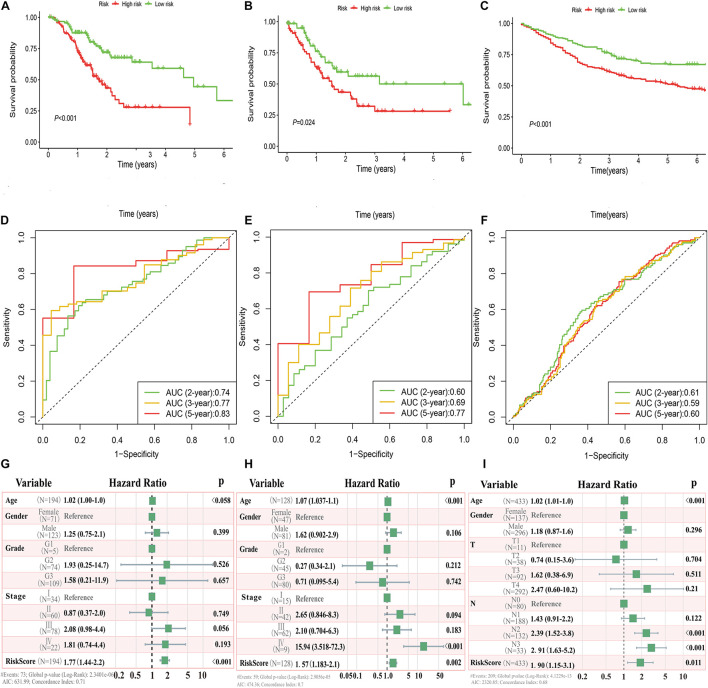
Immune risk signature was associated with GC survival. Kaplan–Meier curves of overall survival based on low- and high-risk groups in the TCGA training cohort **(A)**, TCGA testing cohort **(B)**, and GEO validation cohort **(C)**; receiver operating characteristic (ROC) curve with AUC values for validation of the immune risk signature’s predictive performance in the TCGA training cohort **(D)**, TCGA testing cohort **(E)**, and GEO validation cohort **(F)**; forest plot of the multivariate Cox regression analysis delineated the association between immune risk signature and survival in the three cohorts compared with other clinical variables **(G–I)**, respectively.

In the TCGA and GEO cohorts, we observed significant differences in prognosis among patients in different immune risk groups *via* Kaplan–Meier curves (Figures 3A–C). Moreover, the AUC values of 2-, 3-, and 5-years were 0.74, 0.77, and 0.83 in the training cohort, 0.60, 0.69, and 0.77 in the TCGA testing cohort, and 0.61, 0.59, and 0.60 in the GEO cohort, respectively (Figures 3D–F).

Multivariate analyses were conducted to validate whether this immune signature was the risk factor of GC, not concerned with clinical features (age, gender, grade classification, and tumor stage). The results showed that the risk score was an independent variable related to the survival of GC patients in all three cohorts (*P* < 0.01, Figures 3G–I).

### Correlation Assessment Between Immune Risk Signature and Immune Cell Infiltration

**FIGURE 4:**
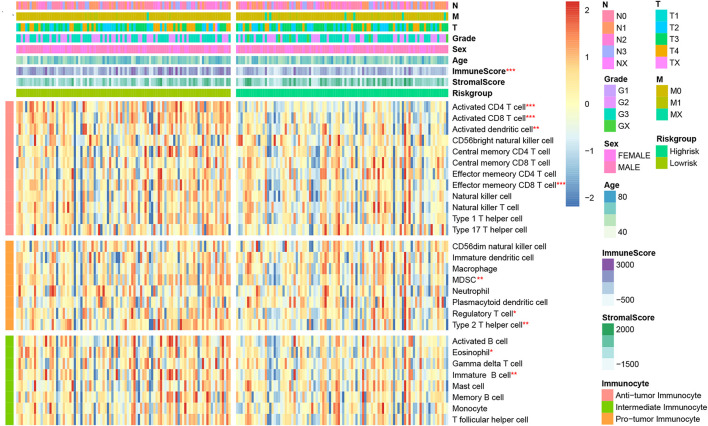
Correlation between immune risk signature and immune cell infiltration. Single-sample gene set enrichment analysis (ssGESA) determined the relative abundance of 28 types of immune cell subpopulations with high- and low-risk signature subgroups. *P < 0.05, **P < 0.01, and ***P < 0.001 (Mann–Whitney–Wilcoxon test).

We would like to further evaluate the relationship between the immune signature and TIM characteristics. Hence, we draw a heatmap by ssGSEA to visualize the relative abundance of 28 immune infiltrating cell subsets from the TCGA training dataset (Figure 4). Obviously, the cell subset of anti-tumor lymphocytes including activated dendritic cells (*P* = 0.009), activated CD4^+^/CD8^+^ T cells (*P* < 0.001), and effector memory CD8 T cells (*P* < 0.001) were enriched in the low-risk signature group. Protumor immunocyte subtypes like MDSC (*P* = 0.002), regulatory T cells (*P* = 0.042), and type 2 T helper cell (*P* = 0.007) and intermediate immunocyte subtypes like eosinophil (*P* = 0.014) and immature B cell (*P* = 0.008) were also elevated in the low-risk signature group. Subsequently, the conclusion was validated again in TCGA and GEO validation datasets (Supplementary Figure 2), and a similar tendency was observed in the two-risk stratification groups. Moreover, immune and stromal scores were counted by the ESTIMATE algorithm. Analysis of differences between groups showed that the immune score of the low-risk group was higher (*P* < 0.001) than that of the high-risk group, but the stromal score was relatively low (Supplementary Figure 3), which was basically in line with the results observed within the entire TCGA and GEO validation groups (*P* < 0.05).

### Functional Enrichment Analysis

**FIGURE 5:**
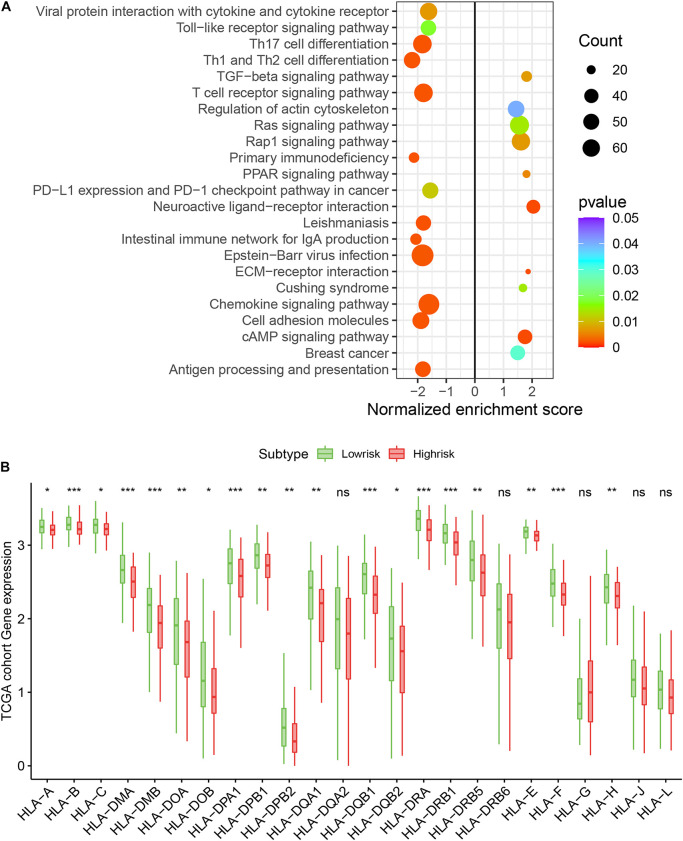
Top enriched signaling pathways in distinct immune risk signature groups from the TCGA training cohort were assessed using the GSEA algorithm **(A)**. The immune risk signature was associated with human leukocyte antigen (HLA) **(B)**. *P < 0.05, **P < 0.01, ***P < 0.001, ^ns^*P* > 0.5.

We assessed the newly identified signature’s roles in regulating the gene enrichment pathways *via* the related biological signaling pathway analysis based on GSEA analysis (Figure 5A). The TGFβ, Ras, and Rap1 pathways involved in tumor proliferation, migration, and invasion were obviously enriched in the immune high-risk cohort. In the immune low-risk signature, Th17/Th1/Th2 differentiation signaling pathways, T cell receptor as well as PD-L1 expression and PD-1 checkpoint pathways were mainly activated.

### Immune Risk Signature Evaluated Immune Response

**FIGURE 6:**
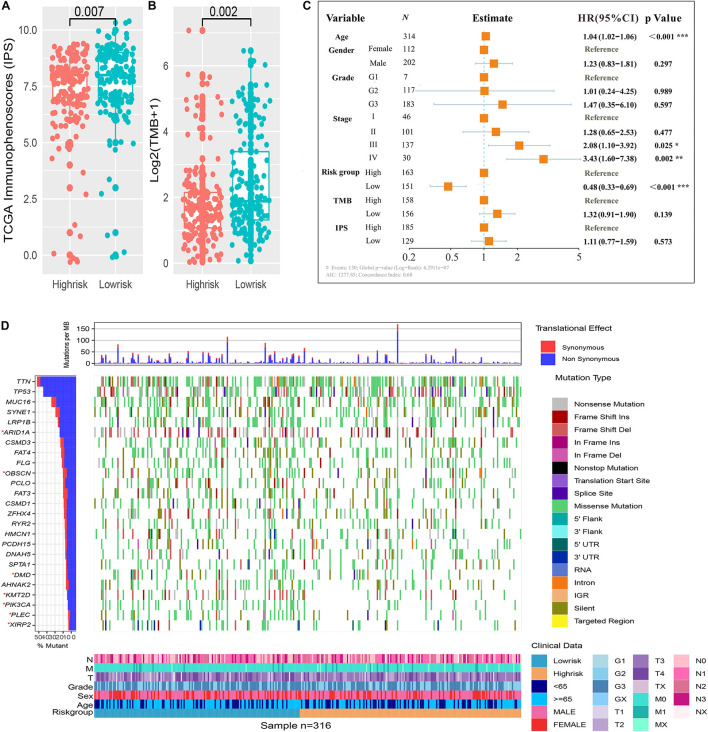
Immune risk signature was associated with Immunophenoscore (IPS) and tumor mutation burden (TMB). Difference boxplot of IPS and TMB between high- and low-risk immune groups **(A,B)**. Multivariate Cox regression analysis **(C)** delineated the survival-related independent factors, taking into account clinical variables, signature, IPS, and TMB. Mutational landscape of SMGs **(D)** stratified by high- and low-risk signature groups was presented based on the somatic mutational annotation files of GC in the TCGA cohort.

We assessed the difference in expression of 24 HLA-related genes in all three datasets. We found that HLA-B, HLA-DMA, HLA-DMB, HLA-DOA, HLA-DOB, HLA-DPA1, HLA-DPB1, HLA-DPB2, HLA-DQA1, HLA-DQB1, HLA-DQB2, HLA-DRA, HLA-DRB1, HLA-DRB5, HLA-E, HLA-F, and HLA-H were elevated (*P* < 0.05) in the immune low-risk signature group, which may indicate a good immune response (Figure 5B). Validation within the entire TCGA and GEO groups was shown in Supplementary Figure 4. According to the results of IPS, the low-risk group had an obviously higher score compared with the high-risk group in the TCGA dataset (*P* = 0.007, Figure 6A).

### Immune Risk Signature Determined the Mutation Landscape

To improve the understanding of the correlation between the newly constructed immune signature and mutational landscape, TMB was counted and summed. Patients in the low-risk group tended to exhibit higher TMB than those in the high-risk group in the TCGA dataset (*P* = 0.002, Figure 6B). Besides, the study further explored the association between survival and various variables such as clinical factors, IPS, TMB, and newly constructed immune signature, which indicated that the immune signature was still an independent survival-related factor (*P* < 0.001, Figure 6C). Significantly mutated genes (SMGs) analysis for GC samples (high-risk vs. low-risk subgroups) was demonstrated in Figure 6D. The SMGs including ARID1A [2 of 164 (1.22%) vs. 12 of 143 (8%), *P* = 0.008], PIK3CA [12 of 164 (7.32%) vs. 33 of 143 (22%), *P* < 0.001], OBSCN [15 of 164 (9.15%) vs. 29 of 143 (19.33%), *P* = 0.015], PLEC [11 of 164 (6.71%) vs. 25 of 143 (16.67%), *P* = 0.01], DMD [14 of 164 (8.54%) vs. 28 of 143 (18.67%), *P* = 0.013], KMT2D [13 of 164 (7.93%) vs. 33 of 143 (22%), *P* < 0.001], and XIRP2 [10 of 164 (6.1%) vs. 20 of 143 (13.33%), *P* = 0.046] were marked in Figure 6D.

### Expression Verification in STAD by qRT-PCR

**FIGURE 7:**
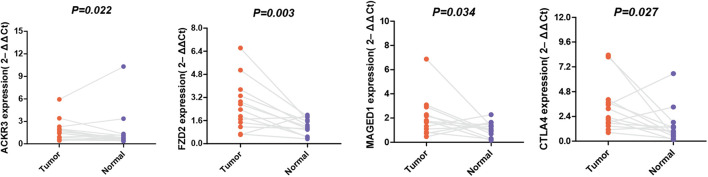
The mRNA expression of MAGED1, ACKR3, FZD2, and CTLA4 in a GC tumor and the adjacent tissues.

The expression levels of these selected genes were tested using qRT-PCR to further prove their reliability. As shown in Figure 7, all the mRNA transcripts were detected and their expressions in STAD were significantly higher than those in adjacent normal tissues (*p* < 0.05).

## Discussion

Tumor immune contexture plays a key role in tumorigenesis, progression, and metastasis (Dunn et al., 2004; Fridman et al., 2012; Remark et al., 2015). ICIs have been regarded as a promising treatment in advanced or metastatic GC. Yet, up to now, only a small number of GC patients have benefited from the ICIs treatment. In this research, we have developed an immune risk signature based on four immune-related genes (MAGED1, FZD2, ACKR3, and CTLA4) in a stand-alone dataset, and have validated its performance in TCGA and GEO datasets. This new signature can stratify GC patients into high- and low-risk subgroups and serve as a potential prognostic biomarker of immunotherapy.

Similar to previous investigation (Lee et al., 2008; Kang et al., 2017), our study indicates that many types of immune cells are enriched in low-risk immune groups, including activated CD4^+^/CD8^+^ T cell, activated dendritic cell, effector memory CD8^+^ T cell, eosinophil, immature B cell, MDSC, regulatory T cell, and type 2 T helper cell, with better survival. Protumor immunocyte types like MDSC and regulatory T cell, as adverse prognostic factors in GC (Ichihara et al., 2003; Wang B. et al., 2011), similarly increase in the low-risk immune group. This inconsistent result may be due to the actual high ratio of anti-tumor immunocytes compared with protumor immunocyte, presenting a favorable factor in GC survival. In addition, stromal and immune scores calculated by the ESTIMATE algorithm showed that immune scores are significantly elevated and stromal scores depressed in the low-risk immune group, which is consistent with studies that emphasize that these patients may be sensitive to immunotherapy (Mao et al., 2013; Mariathasan et al., 2018). The GSEA analysis revealed that TGF-β, Ras, and Rap1 signaling pathways are easily activated in high-risk signature and regulate tumorigenesis, proliferation, and invasion, which has been confirmed in the previous studies (Adjei, 2001; Tauriello et al., 2018; Liu et al., 2021). Other pathways activated in low-risk signature like Th17/Th1/Th2 differentiation, T cell receptor signaling as well as PD-L1 expression and PD-1 checkpoint pathways potentially improve the therapeutic efficacy (Thorsson et al., 2018). The new immune signature helps to screen high-risk population and facilitate the development of precision immunotherapy.

The validation analysis reveals that the immune-related signature performs well in therapeutic and prognostic prediction for GC, and low-risk patients are characterized as higher mutation burden and expression level of HLA genes. HLA, often referred to as the MHC molecule or major histocompatibility complex, varies greatly from person to person. In addition, it is a marker for mutually recognizing the immune cells of different individuals, which participates in the immune response and has very important biological functions. HLA-I (MHC-I), of great importance, consists of a highly polymorphic α-heavy chain and a β2-microglobulin (β2M) light chain, encoded by the HLA-A, HLA-B, or HLA-C genes, delivers peptides to CD8^+^ T cells, and is essential for immune surveillance and cancer immunotherapy (Hazini et al., 2021). Patients with increased expression of HLA-A, HLA-B, or HLA-C genes have a higher efficiency of antigen delivery. HLA-I dysregulation in cancer patients results in poor immune outcomes. Shim et al. (2020) have shown that correcting HLA can improve the immune efficacy of NSCLC. In our study, the immune-related risk score was able to assess patients with different levels of HLA-A, HLA-B, or HLA-C expression, thus guiding the use of immunotherapy to some extent. High TMB is often regarded as a sensitive marker of immune-checkpoint blockade, particularly a high mutation proportion of driver genes (Rizvi et al., 2015; Samstein et al., 2019). The SMGs like ARID1A, PIK3CA, OBSCN, PLEC, DMD, KMT2D, and XIRP2 are enriched in low-risk groups. ARID1A and PIK3CA have significant association with microsatellite instability (Wang K. et al., 2011; Mathur, 2018; Shen et al., 2019), predicting good response for immunotherapy of GC. DMD acts as a favorable gene for survival of gastric adenocarcinomas (Jones et al., 2020). The relationship between other SMGs and immunotherapy have not been reported yet. Moreover, IPS also exhibits a rising tendency in low-risk immune groups. To investigate the survival-related covariates, we included TMB, IPS, and other clinical factors into multivariate analysis, and found that this signature remains an independent prognostic factor. Therefore, it is reasonable to think that people with a low-risk immune systems may probably gain clinical benefits from checkpoint immunotherapy. The new immune signature can distinguish immune status and promote the progress of precision immunotherapy.

There are still several limitations in our study. For one, immune-related features are only constructed based on public datasets, which have not been confirmed in clinical cohorts. Prospective clinical studies involving immunotherapy combined with basic experiments are needed for further validation. For another, as the lack of mutational profile in the GEO database, the mutational landscape is not validated in independent datasets. In addition, the AUC values of the above prognostic signature cannot be very high, and some AUC values are only at fair levels, especially in the GEO independent validation group. This is because our study only extracted immune-related genes for model construction. So far, studies have identified other gene categories closely linked to tumors and built models accordingly, such as ferroptosis-related genes and m6A-related genes. A mature precision model with strong AUC value level for gastric cancer is bound to be an integrated model through various genomics, proteomics, and even radiomics, which is also bound to be a long process of effort. Despite this, the findings of our study still have noteworthy implications for survival prediction along with the evaluation of precision immunotherapy for GC patients.

In a word, our study has developed a new immune-related signature that could not only predict the prognosis of GC patients but also reveal intratumor immune response, which may guide the development of novel treatment strategies combining chemotherapy, targeted therapy, and immunotherapy.

## Data Availability Statement

Publicly available datasets were analyzed in this study. This data can be found here: Publicly available datasets were analyzed in this study. This data can be found here: TCGA (https://gdc.cancer.gov/) and GEO database (https://www.ncbi.nlm.nih.gov/geo/).

## Ethics Statement

Ethical review and approval was not required for the study on human participants in accordance with the local legislation and institutional requirements. Written informed consent for participation was not required for this study in accordance with the national legislation and the institutional requirements.

## Author Contributions

FL and TR: conception and design and manuscript review. TL and SD: methodology development, data collection, and manuscript writing. TL, SD, X-QL, X-YL, and KX: data analysis and interpretation. All authors approved the final manuscript.

## Conflict of Interest

The authors declare that the research was conducted in the absence of any commercial or financial relationships that could be construed as a potential conflict of interest.

## Publisher’s Note

All claims expressed in this article are solely those of the authors and do not necessarily represent those of their affiliated organizations, or those of the publisher, the editors and the reviewers. Any product that may be evaluated in this article, or claim that may be made by its manufacturer, is not guaranteed or endorsed by the publisher.

## Footnotes

1
https://immport.niaid.nih.gov

## Supplementary Material

Supplementary Figure 1 Expression heat map, survival status, and risk score of the signature in the TCGA testing cohort and GEO validation cohort were depicted.

Supplementary Figure 2 Estimation of the relative proportion of 28 types of immune cell subpopulations stratified by high- and low-risk signature groups in the TCGA testing cohort and GEO validation cohort. ^∗^*P* < 0.05, ^∗∗^*P* < 0.01, ^∗∗∗^*P* < 0.001 (Mann–Whitney–Wilcoxon test).

Supplementary Figure 3 Difference of immune scores and stromal scores between low- and high-risk immune groups in the TCGA training cohort, TCGA testing cohort, and GEO validation cohort.

Supplementary Figure 4 Immune risk signature was associated with human leukocyte antigen (HLA) in the entire TCGA and GEO cohort.

## Abbreviations

GC: gastric cancer
STAD: stomach cancer
ICIs: immune checkpoint inhibitors
MSI: microsatellite-instability
IPS: Immunophenoscore
TMB: tumor mutation burden
TIM: tumor immune microenvironment
MDSC: myeloid-derived suppressor cells
Lasso regression analysis: the least absolute shrinkage and selection operator regression analysis
AUCs: area under the ROC curves
ssGSEA: single sample gene set enrichment analysis
SMGs: significantly mutated genes
GSEA: gene set enrichment analysis
KEGG: Kyoto Encyclopedia of Genes and Genomes
IRGs: immune-related genes
HLA: human leukocyte antigen
MHC: major histocompatibility complex.
