# Supplementary figures and images for: Identification of an Immune-Related Signature Predicting Survival Risk and Immune Microenvironment in Gastric Cancer
Source: Front Cell Dev Biol. 2021 Nov 2;9:687473. doi: 10.3389/fcell.2021.687473 (PMC8596572; doi:10.3389/fcell.2021.687473)

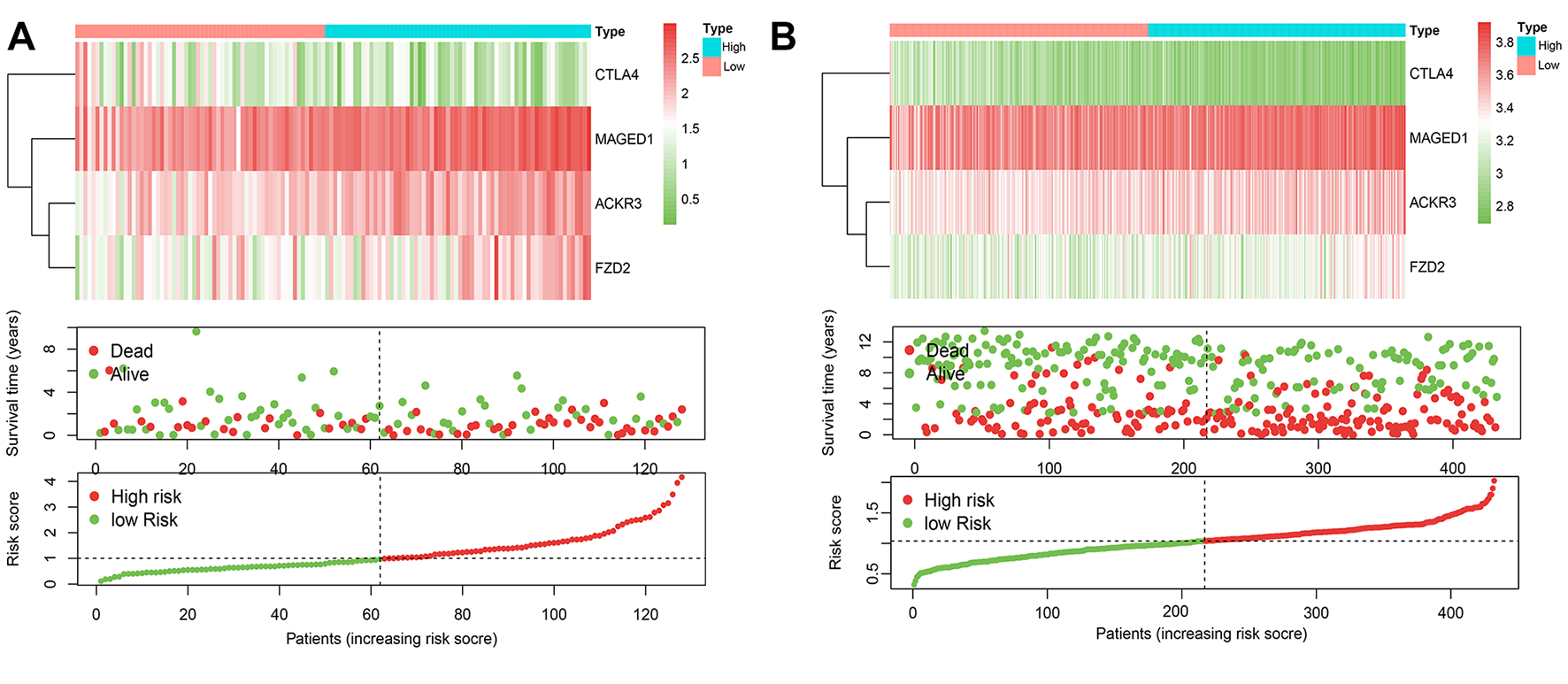

Supplement: Supplementary Figure 1 — Expression heat map, survival status, and risk score of the signature in the TCGA testing cohort and GEO validation cohort were depicted. [file Image_1.TIF]

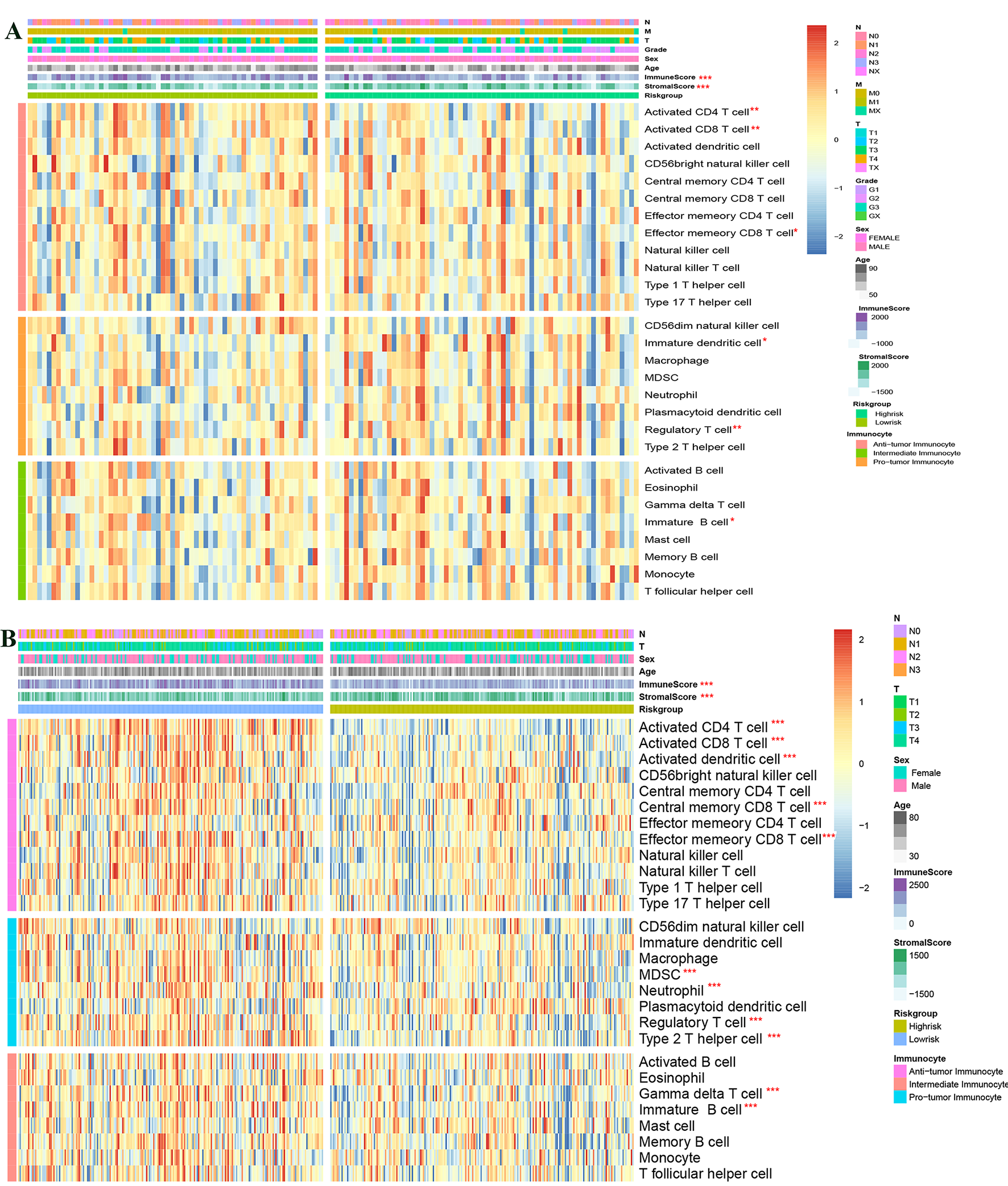

Supplement: Supplementary Figure 2 — Estimation of the relative proportion of 28 types of immune cell subpopulations stratified by high- and low-risk signature groups in the TCGA testing cohort and GEO validation cohort. ∗P < 0.05, ∗∗P < 0.01, ∗∗∗P < 0.001 (Mann–Whitney–Wilcoxon test). [file Image_2.TIF]

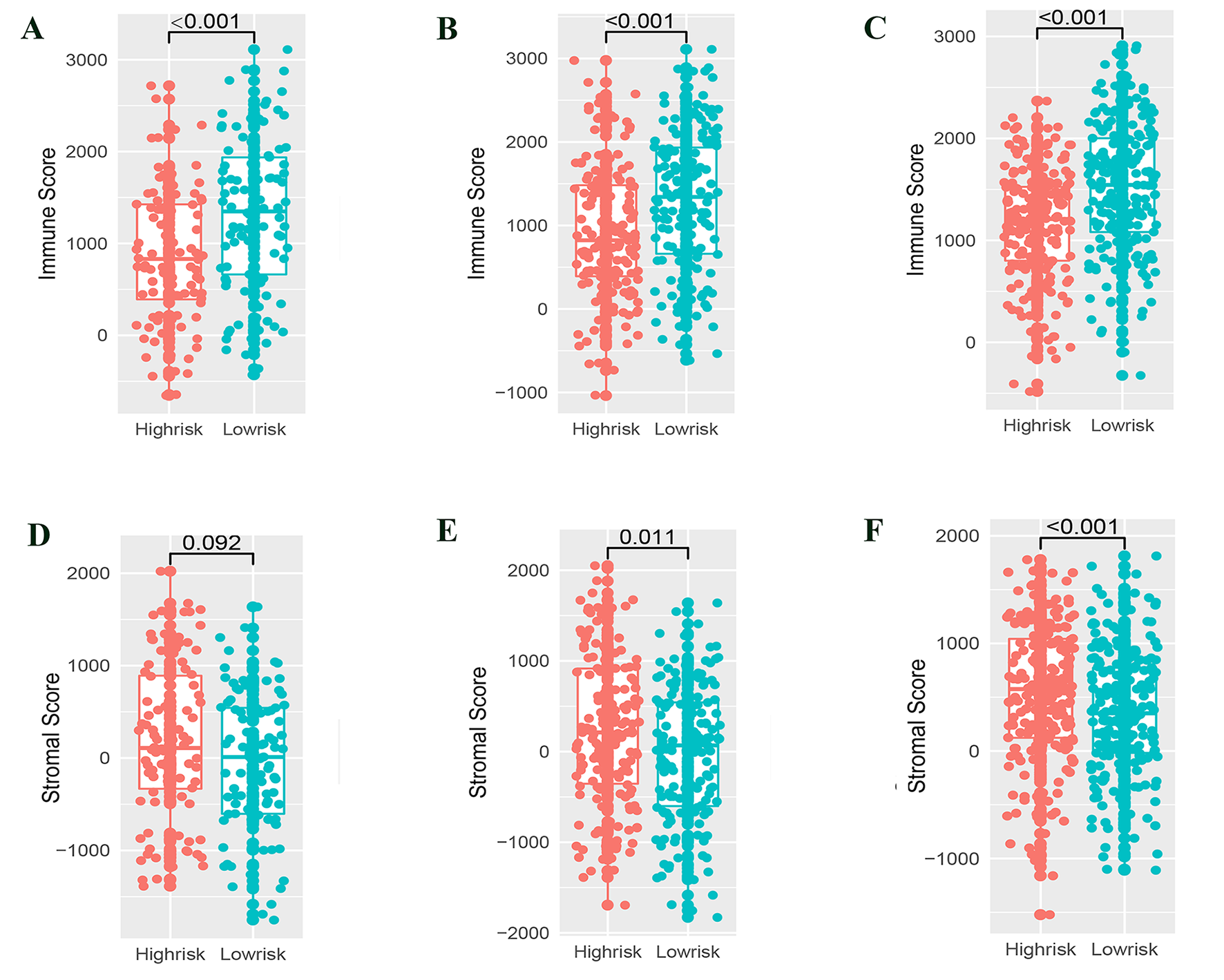

Supplement: Supplementary Figure 3 — Difference of immune scores and stromal scores between low- and high-risk immune groups in the TCGA training cohort, TCGA testing cohort, and GEO validation cohort. [file Image_3.TIF]

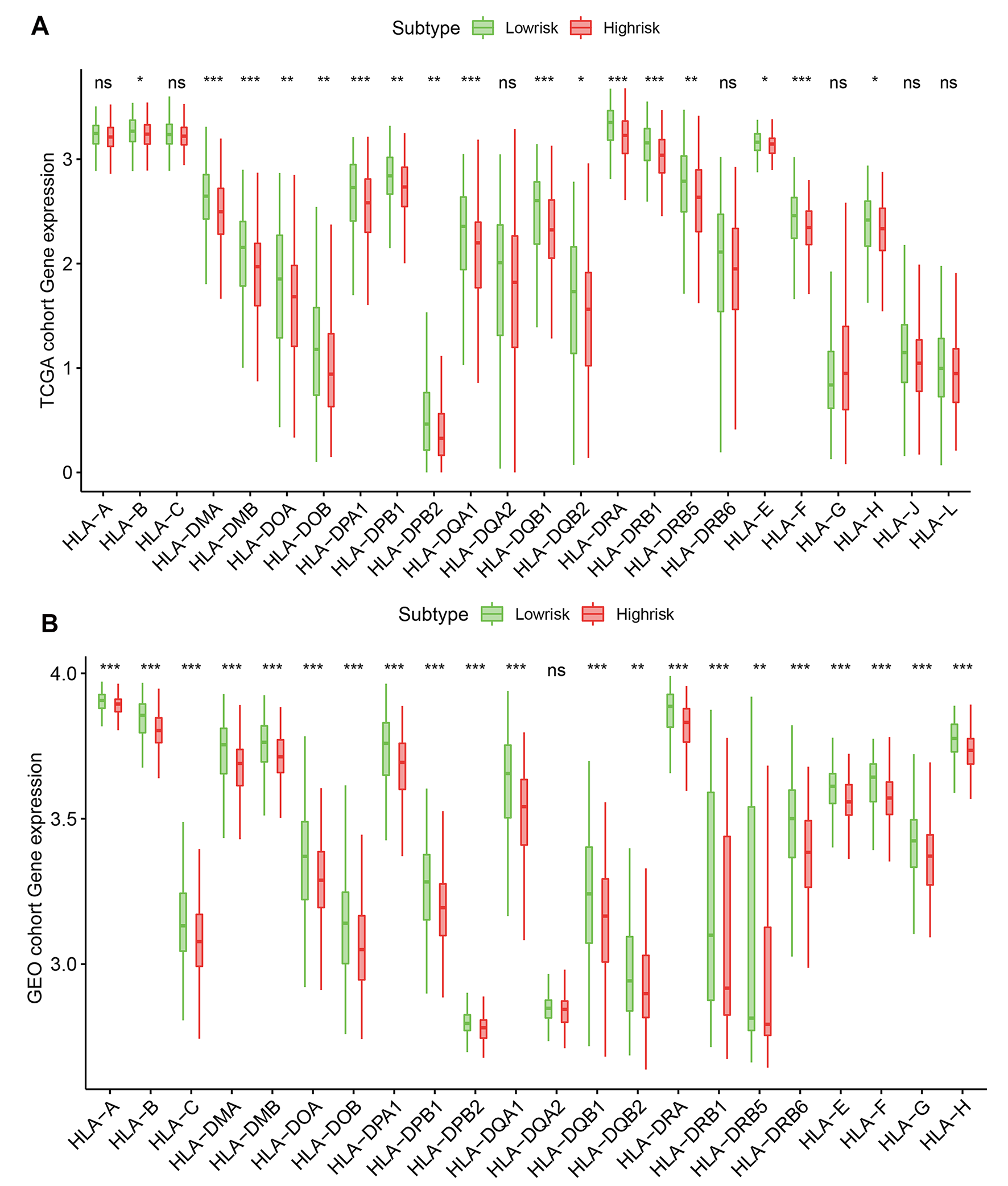

Supplement: Supplementary Figure 4 — Immune risk signature was associated with human leukocyte antigen (HLA) in the entire TCGA and GEO cohort. [file Image_4.TIF]
